# The double-edged sword role of hydrogen sulfide in hepatocellular carcinoma

**DOI:** 10.3389/fphar.2023.1280308

**Published:** 2023-10-11

**Authors:** Huijie Zhao, Yanting Zhang, Xiaodi Fu, Chaoren Chen, Saadullah Khattak, Honggang Wang

**Affiliations:** ^1^ Institute of Chronic Disease Risks Assessment, Henan University, Kaifeng, China; ^2^ School of Basic Medical Sciences, Henan University, Kaifeng, Henan, China; ^3^ School of Clinical Medicine, Henan University, Kaifeng, Henan, China; ^4^ School of Nursing and Health, Institute of Nursing and Health, Henan University, Kaifeng, Henan, China; ^5^ Henan International Joint Laboratory for Nuclear Protein Regulation, School of Basic Medical Sciences, Henan University, Kaifeng, Henan, China; ^6^ School of Life Sciences, Henan University, Kaifeng, China

**Keywords:** hydrogen sulfide, hepatocellular carcinoma, apoptosis, cystathione gamma-lyase, 3-mercaptopyruvate sulfurtransferase

## Abstract

With an increasing worldwide prevalence, hepatocellular carcinoma (HCC) is the most common primary malignant tumor of the liver in the world. It is also the primary reason for cancer-related death in the world. The pathogenesis of HCC is complex, such as DNA methylation changes, immune regulatory disorders, cell cycle disorders, chromosomal instability, and so on. Although many studies have been conducted on HCC, the molecular mechanisms of HCC are not completely understood. At present, there is no effective treatment for HCC. Hydrogen sulfide (H_2_S) has long been regarded as a toxic gas with the smell of rotten eggs, but recent studies have shown that it is an important gasotransmitter along with carbon monoxide (CO) and nitric oxide (NO). Increasing evidence indicates that H_2_S has multiple biological functions, such as anti-inflammation, anti-apoptosis, anti-oxidative stress, and so on. Recently, a lot of evidence has shown that H_2_S has a “double-edged sword” effect in HCC, but the mechanism is not fully understood. Here, we reviewed the progress on the role and mechanism of H_2_S in HCC in recent years, hoping to provide a theoretical reference for future related research.

## 1 Introduction

Hepatocellular carcinoma (HCC) is an important primary liver cancer and a serious medical problem in the world. At present, HCC has been regarded as the leading cause of death of patients with liver cirrhosis, and its incidence rate is expected to increase in the future (Forner et al., 2018; Sim and Knox, 2018; Llovet et al., 2021). The evidence indicates that by 2025, about 1 million people will be affected by HCC every year. More than 90% of HCC cases occur in the environment of chronic liver diseases (Renne et al., 2021). The main risk factors of HCC include diabetes, alcoholism, chronic hepatitis, nonalcoholic fatty liver disease (NAFLD) and exposure to dietary toxins, such as aflatoxin and aristolochic acid (Kulik and El-Serag, 2019; Yang et al., 2019; Gilles et al., 2022). The pathogenesis of HCC is complex, and involves a variety of molecular faults, including DNA methylation change, immune regulation disorder, cell cycle disorder, chromosome instability, epithelial cell to mesenchymal cell transition (EMT), microRNA (miRNA) disorder, and the increased HCC stem cells (Chidambaranathan-Reghupaty et al., 2021). If diagnosed early, HCC may be cured and have a good long-term prognosis. However, the vast majority of HCC patients are found in the late stage. At this time, the surgical treatment is no longer a choice (Dimitrou et al., 2017). Instead, it requires chemotherapy, using chemicals to destroy cancer cells and inhibit the proliferation of new cancer cells (Chang et al., 2020). Therefore, it is particularly important to find suitable chemotherapy drugs for HCC.

**FIGURE 1:**
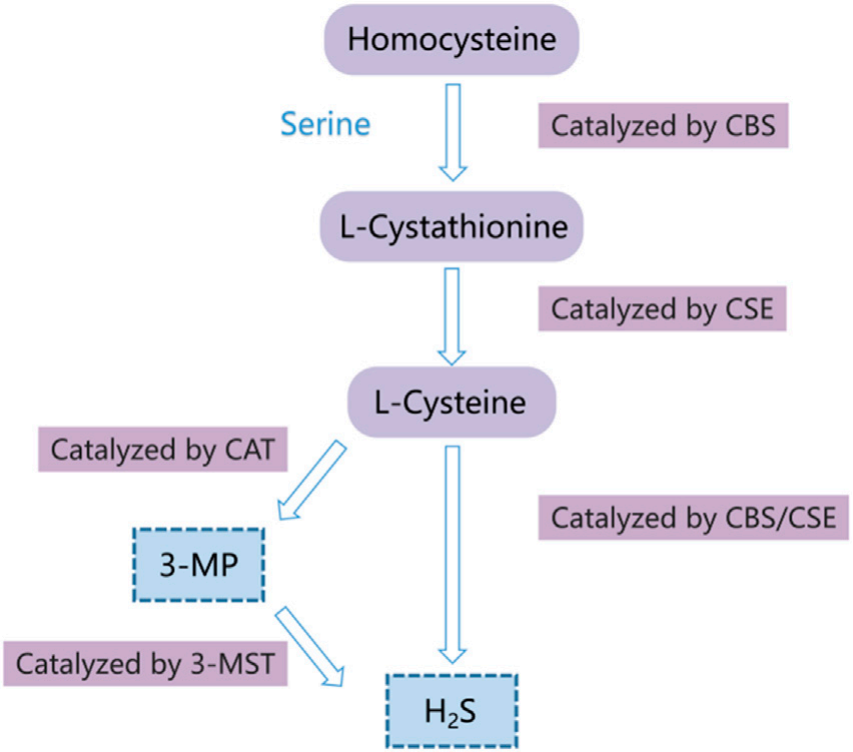
Diagram of the endogenous H_2_S generation process. CSE, cystathionine-gamma-lyase; 3-MST, 3-mercaptopyruvate thiotransferase; CAT, cysteine aminotransferase; CBS, cystathionine-beta-synthase; 3-MP, 3-mercaptopyruvate.

Hydrogen sulfide (H_2_S) has long been considered as a toxic gas with the rotten egg odor. However, it was regarded as the third gaseous signal molecule after carbon oxide (CO) and nitric oxide (NO) recently (Powell et al., 2018; Zaorska et al., 2020). Currently, there are mainly three enzymes that catalyze endogenous H_2_S production, namely, cystathionine gamma-lyase (CSE), 3-mercaptopyruvate sulfurtransferase (3-MST), and cystathionine-beta-synthase (CBS) (Coavoy-Sánc et al., 2020; Dilek et al., 2020; Shackelford et al., 2021a). During endogenous H_2_S production, CBS catalyzes the β-substitution reaction of homocysteine with serine to generate cystathionine. Cysteine is produced through α, γ-cysteine elimination of cystathionine catalyzed by CSE. Cysteine can be transformed into H_2_S via the β-elimination reaction under the catalysis of CBS and CSE. 3-mercaptopyruvate (3-MP) is formed via transferring amines from cystine into α-ketoglutarate catalyzed by cysteine aminotransferase (CAT). 3-MST catalyzes 3-MP sulfur to produce H_2_S (Figure 1) (Wang et al., 2020a; Casin and Calvert, 2021; Zhao et al., 2021). The mechanisms of the effects of H_2_S on cell functions mainly include regulation of the activity of transcription factors, histone modification, DNA damage repair, DNA methylation, and post-translational modification of proteins through the sulfur hydration of H_2_S (Dongó et al., 2018). The evidence indicates that H_2_S plays a vital role in multiple pathological and physiological processes, such as anti-inflammation (Mohammed et al., 2021), anti-apoptosis (Fouad et al., 2020), anti-oxidative stress (Tocmo and Parkin, 2019), blood pressure reduction (Greaney et al., 2017; Zaorska et al., 2019), and the regulation of cell survival/death, cell proliferation/hypertrophy and cell differentiation (Zhang et al., 2017). Hence, H_2_S participates in many diseases, such as lung diseases (Pacitti et al., 2021), ischemia/reperfusion injury (Krylatov et al., 2021), and cancers (Shackelford et al., 2021b; Khattak et al., 2022; Faris et al., 2023). In recent years, many studies have revealed that H_2_S has the dual effects of anticancer and cancer promotion in HCC, but the relevant mechanisms are not completely understood. Hence, we summarized the recent studies on the role and mechanisms of H_2_S in HCC through PubMed, hoping to provide a theoretical reference for future related research.

## 2 H_2_S inhibits hepatocellular carcinoma

### 2.1 Exogenous H_2_S inhibits hepatocellular carcinoma

#### 2.1.1 Exogenous H_2_S inhibits hepatocellular carcinoma by blocking the STAT3 pathway

The signal transducer and activator of transcription (STAT) protein is a potential cytoplasmic transcription factor, which includes seven members: STAT1, STAT2, STAT3, STAT4, STAT5a, STAT5b, and STAT6 (Loh et al., 2019). STAT3, a main sensor that mediates the signal transmission of interleukin-6 (IL-6) to the nucleus, participates in cell growth, regeneration, survival, differentiation, immune responses, and cell respiration. The STAT3 activation is strictly regulated in normal tissues. However, the abnormal activation of STAT3 is related to the formation, progression, and metastasis of cancers (El-Tanani et al., 2022; Sadrkhanloo et al., 2022). The relevant mechanism remains to be clarified. The results of SEN LU et al. showed that GYY4137(a donor of H_2_S) suppressed IL-6-induced STAT3 activation through effectively decreasing p-STAT3 levels by reducing JAK2 phosphorylation (an activator of STAT3) in HCC cells. GYY4137 also reduced the expression levels of STAT3 downstream proteins, including cyclin D1, Bcl-2, myeloid cell leukemia sequence 1 (Mcl-1) and survivin. The number of HCC cells in the G0/G1 phase of the cell cycle was increased by GYY4137, which was consistent with GYY4137 inhibition of cyclin D1, suggesting that cell cycle arrest of HCC cells was induced by exogenous H_2_S. GYY4137 promoted the cleavage of poly (ADP-ribose) polymerase (PARP) and upregulated the levels of cleaved caspase-9 and caspase-3 in HCC cells, suggesting that exogenous H_2_S induced apoptosis of HCC cells, which was consistent with the inhibition of GYY4137 on Bcl-2, survivin, and Mcl-1. In addition, GYY4137 suppressed HCC cell’s viability time- and dose-dependently and suppressed the angiogenesis by downregulating the levels of vascular endothelial growth factor (VEGF) and hypoxia-inducible factor-1α (HIF-1α) that were STAT3 downstream proteins. Similar to the results *in vitro*, GYY4137 notably suppressed tumor growth in the model of subcutaneous HCC cells xenotransplantation through suppressing the activation of STAT3 and its downstream target gene expression *in vivo*. Hence, it could be inferred that exogenous H_2_S inhibited the proliferation, metastasis and invasion of HCC through inducing cell cycle arrest and apoptosis of HCC cells, and the suppression of the angiogenesis by suppressing the STAT3 pathway (Lu et al., 2014), which is consistent with the fact that the abnormal activation of STAT3 promotes tumor cell proliferation via increasing cyclin D1 level, and inhibits apoptosis via increasing the levels of Bcl-2, survivin, and Mcl-1 (Garcia et al., 2001). In the above study, how H_2_S inhibits JAK phosphorylation through its sulfur hydration to reduce STAT3 phosphorylation level needs to be further clarified. STAT3 pathway will become a vital target for H_2_S-related drugs to treat HCC.

#### 2.1.2 Exogenous H_2_S suppresses hepatocellular carcinoma by promoting autophagy

Besides apoptosis, cell cycle arrest and angiogenesis, autophagy is also involved in anti-tumor effects (Ahmed et al., 2022). Moreover, H_2_S plays an important role in cancers by regulating autophagy (Iqbal et al., 2021). Therefore, it is natural to speculate that H_2_S may inhibit HCC by regulating autophagy. Autophagy is a homeostatic process, in which cell components and structures are transferred to lysosomes for degradation and recycling. It can also remove the waste materials from cells, including the damaged organelles and protein aggregation, and help to clear the invading pathogens. From yeast to mammals, the mechanism of autophagy is conservative (Ganzleben et al., 2021; Kumar et al., 2021; Zahedi-Amiri et al., 2021). The disorder of autophagy is often related to the pathogenesis of various cancers, which not only inhibits the cancer but also promotes the cancer (Devis-Jauregui et al., 2021; Rakesh et al., 2022). To study the role and mechanism of exogenous H_2_S in HCC by regulating autophagy, Shanshan S Wang and colleagues committed a lot of experiments, and the results revealed that NaHS treatment increased the expressions of Atg5 and LC3-II, and decreased p62 expression in HCC cells. The transmission electron microscopy showed that the number of intracellular double-membrane vesicles increased in NaHS-treated HCC cells. This indicated that exogenous H_2_S upregulated autophagy in HCC cells (Wang et al., 2017). It has been reported that rapamycin, an inhibitor of the mechanistic target of rapamycin (mTOR) and an activator of autophagy, induces autophagy via suppressing protein kinase B (AKT)/phosphatidylinositol-3-kinase (PI3K)/mTOR pathway (Sundarraj et al., 2021; Kamel et al., 2022). Like rapamycin, NaHS also notably downregulated the levels of p-PI3K, p-Akt and mTOR proteins in HCC cells. Moreover, the treatment of NaHS combined with rapamycin further upregulated autophagy, indicating that exogenous H_2_S promoted autophagy by suppressing the PI3K/AKT/mTOR pathway. In addition, exogenous H_2_S suppressed the proliferation, cell cycle progression and migration of HCC cells, but induced apoptosis of HCC cells, which was enhanced by rapamycin. Summarily, exogenous H_2_S suppressed the migration and proliferation of HCC cells via inducing apoptosis and cell cycle arrest through inducing autophagy by suppressing the PI3K/AKT/mTOR pathway (Wang et al., 2017). In addition to the PI3K/AKT/mTOR pathway, our previous study demonstrated that exogenous H_2_S upregulated autophagy via the AMPK/mTOR pathway. In HCC (Wang et al., 2019), whether exogenous H_2_S regulates autophagy through other signal pathways, such as the AMPK/mTOR pathway, needs further study. Contrary to some of the above conclusions that exogenous H_2_S ameliorates HCC by activating autophagy, exogenous H_2_S improves liver diseases and nervous system diseases by inhibiting autophagy-mediated cell death (Nguyen et al., 2021a; Nguyen et al., 2021b). The reason may be related to the differences in the type and course of diseases, the type of tissue cells and the base level of autophagy of cells, which needs to be further studied.

#### 2.1.3 Exogenous H_2_S enhances doxorubicin sensitivity to hepatocellular carcinoma cells by inhibiting the outflow of doxorubicin

One of the most vital limitations of cancer chemotherapy is that the anti-cancer response of cancer patients decreases over the extended treatment period. This phenomenon is called multidrug resistance (MDR), which is the primary reason for cancer treatment failure (Nikolaou et al., 2018; Wang et al., 2021a). MDR is associated with drug efflux, particularly through many membrane-binding proteins named ATP binding cassette (ABC) transporters. The overexpression of these proteins decreases the accumulation of chemotherapy drugs in cells, which may contribute to the MDR of some cancers (Gupta et al., 2018; Kopecka et al., 2020). Therefore, it is very important to reduce the drug resistance of cancer cells to improve the anti-cancer efficacy. Eric Stokes and colleagues found that doxorubicin reduced the expression of endogenous H_2_S-producing enzyme (CSE) in HCC cells. Exogenous H_2_S promoted doxorubicin inhibition of colony formation and cell survival, while exogenous H_2_S alone didn’t have this effect. Additionally, exogenous H_2_S promoted the cellular accumulation of doxorubicin through inhibiting the levels of ABCA1 and ABCG8, which is the underlying mechanism of the synergistic effect of doxorubicin and H_2_S. Moreover, exogenous H_2_S notably inhibited the heterodimer formation between retinoid X receptor beta (RXRβ) and liver X receptor alpha (LXRα) induced by doxorubicin and weakened the binding of LXRα/RXRβ with the promoter of ABCG8 and ABCA1 genes. Exogenous H_2_S S-sulfhydrated RXRβ but not LXRα, and the inhibition of RXRβ S-sulfhydration alleviated H_2_S inhibition of LXRα/RXRβ heterodimer formation. Collectively, it could be deduced that exogenous H_2_S reversed doxorubicin resistance of HCC through inhibiting the levels of ABCA1 and ABCG8 by inhibiting the binding of LXRα/RXRβ with ABCG8 and ABCA1 genes promoter via suppression of the heterodimer formation between RXRβ and LXRα, which needed to be further confirmed (Stokes et al., 2018). The above study indicates that doxorubicin downregulates CSE expression in HCC cells, which may be due to its characteristics of DNA embedding (AbuHammad and Zihlif, 2013). The exact mechanism of doxorubicin regulating CSE needs to be further clarified. In addition, the above study shows that H_2_S S-sulfhydration of RXRβ is the mechanism of reversing MDR of doxorubicin in HCC. It has been reported that the EMT pathway is related to the occurrence of MDR (Erin et al., 2020; Shome and Ghosh, 2021). Therefore, whether H_2_S can regulate tumor MDR through the EMT pathway is required to be further studied in the future. It has been reported that H_2_S inhibits cisplatin resistance of cancer through inducing apoptosis, blocking cell cycle, and suppressing cell migration and invasion (Ma et al., 2018). Hence, whether exogenous H_2_S reversed doxorubicin resistance of HCC through inducing apoptosis, blocking cell cycle, or suppressing cell migration and invasion needs to be studied.

#### 2.1.4 HA-ADT, a novel donor of hydrogen sulfide, suppresses hepatocellular carcinoma

Given the effective inhibitory properties of exogenous H_2_S on HCC mentioned above, H_2_S-related drugs are expected to become new drugs for HCC treatment. At present, the existing H_2_S release agents cannot completely satisfy the requirements of scientific research and clinical trials. Therefore, developing efficient and safe H_2_S release agents is crucial for the clinical application of H_2_S-related drugs. Hyaluronic acid (HA) is a biopolymer that is widely used in many biomedical applications because of its good safety profile, such as drug delivery and tissue engineering (Jung et al., 2014). Methyl derivatives of 5 - (4-hydroxyphenyl) - 3H-1,2-dithio-3-thione (ADT-OH) can be used as H_2_S-releasing agents to produce H_2_S through mitochondrial enzyme metabolism (da Costa et al., 2022; Montoya and Pluth, 2016). HA-ADT, a new type of H_2_S slow-release agent, is a new conjugate formed by connecting HA and ADT-OH through a chemical reaction (Dong et al., 2019). Shao Feng Duan and colleagues studied the effect of HA-ADT on HCC and found that compared to NaHS (a rapidly releasing H_2_S donor) and GYY4137 (a slowly releasing H_2_S donor), HA-ADT exhibited stronger suppression of the proliferation, invasion, and cell cycle progress and migration of human HCC cells. In addition, HA-ADT-induced apoptosis was evidenced by the downregulation of the expressions of p-glycogen synthase kinase-3β (GSK-3β), phospho (p)-protein kinase B (PKB/AKT) and p-β-catenin, and suppressed autophagy through decreasing the expressions of transforming growth factor-β (TGF-β) and p-Smad2/p-Smad3 in human HCC cells. In addition, HA-ADT was more effective in inhibiting the proliferation of liver cancer xenograft tumors than GYY4137 and NaHS. Collectively, HA-ADT inhibited HCC via promoting apoptosis through suppressing GSK-3β/AKT/β-catenin and inhibiting autophagy through suppressing TGF-β/Smad2/3 pathways (Duan et al., 2023). The evidence indicates that exogenous H_2_S suppresses urothelial carcinoma cell proliferation through inducing cell autophagy and apoptosis (Panza et al., 2022), which is inconsistent with the above study that exogenous H_2_S downregulates autophagy to inhibit HCC. This indicates that autophagy may have different activities in different tumors, and plays a dual role in promoting and inhibiting tumor development, according to the cellular environment.

### 2.2 Endogenous H_2_S inhibits hepatocellular carcinoma

Besides exogenous H_2_S, endogenous H_2_S also participates in inhibiting HCC. 3-MST is an important enzyme that catalyzes the production of endogenous H_2_S (Rao et al., 2022). It is located in the vascular endothelium and releases H_2_S rapidly under different stimuli (Zhang et al., 2020). The evidence indicates that 3-MST may be a tumor suppressor and participates in HCC (Li et al., 2022a). However, the mechanism is not completely understood. Meng Li and colleagues found that compared with matched non-tumor tissues, 3-MST expression was notably downregulated in human HCC tissues. The low 3-MST expression was closely associated with the larger tumor size and the lower survival rate. In HCC patients, the higher expression of 3-MST is associated with better clinical outcomes. *3-MST* overexpression in HCC cells suppressed cell proliferation and promoted apoptosis, and also notably restrained the proliferation of tumor xenografts in nude mice. Conversely, the silencing of 3-MST by intratumoral siRNA significantly promoted the growth of HCC. Furthermore, *3-MST* gene knockout aggravated HCC in mice. These outcomes indicated that 3-MST inhibited HCC. *3-MST* overexpression significantly decreased H_2_S level, while siRNA-mediated 3-MST downregulation increased H_2_S level in HCC cells, indicating that endogenous H_2_S production was involved in 3-MST inhibition of HCC. The in-depth research revealed that 3-MST inhibited the HCC cell cycle through suppressing AKT/forkhead box transcription factor 3a (FOXO3a)/retinoblastoma (Rb, an important transcription inhibitor for G1-S progress) signaling pathway (Li et al., 2022b). As we all know, Rb affects tumor progression by regulating apoptosis (Li et al., 2019; Wang et al., 2020b). In the above study, 3-MST negatively regulates Rb, which may result in the induction of apoptosis of HCC cells. The specific mechanism of 3-MST regulating Rb needs further study. Summarily, 3-MST/H_2_S inhibits HCC through promoting the apoptosis and cell cycle arrest of HCC cells by inhibiting the AKT/FOXO3a/Rb pathway (Li et al., 2022b). In addition, in the above study, 3-MST negatively regulates the production of H_2_S in HCC cells. The reason may be the negative feedback between the 3-MST and the CBS/CSE system of H_2_S, which may also be why CSE/H_2_S promotes HCC, while 3-MST/H_2_S has the opposite effect.

## 3 H_2_S promotes hepatocellular carcinoma

### 3.1 Exogenous H_2_S promotes hepatocellular carcinoma

Contrary to the above study, exogenous H_2_S can also promote HCC. STAT3 has been found to be activated to promote the occurrence of HCC (Lee and Cheung, 2019). The results of Yulan Zhen et al. showed that the treatment of HCC cells with NaHS significantly increased the expression levels of p-STAT3 and *STAT3* mRNA, which led to the increased expression levels of COX-2 and *COX-2* mRNA, the increased VEGF level, the reduced cleaved caspase-3 level, the increased viability and migration of HCC cells and the reduction of HCC cells apoptosis. This indicated that exogenous H_2_S promoted HCC by increasing the migration and proliferation and lessening HCC cell apoptosis. While the treatment of HCC cells with AG490 (a STAT3 inhibitor) or NS-398 (a COX-2 inhibitor) notably abolished the above effects of NaHS. Moreover, the treatment of HCC cells with AG490 significantly weakened the increased COX-2 expression induced by NaHS. Meanwhile, the treatment of HCC cells with NS-398 suppressed the increased p-STAT3 expression induced by NaHS. Collectively, exogenous H_2_S aggravated HCC by promoting the proliferation and migration of HCC cells through inhibiting apoptosis and increasing angiogenesis via inducing the STAT3-COX-2 pathway (Zhen et al., 2018). These results provide a new insight into the molecular mechanisms underlying H_2_S promotion of the cell proliferation of HCC cells. Further, the conditions under which exogenous H_2_S inhibits HCC, including inhibiting the migration and proliferation of HCC cells and facilitating HCC cell apoptosis, and on the contrary, the conditions under which exogenous H_2_S promotes HCC, need to be clarified. It can be inferred from a previous study that the low concentration of exogenous H_2_S can promote HCC, while the high concentration of exogenous H_2_S inhibits HCC. The above speculation was confirmed by the experiments of Dongdong Wu and colleagues. Their results showed that H_2_S in human HCC cells was increased compared to that in L02 cells (a kind of human normal hepatocyte), indicating that H_2_S was related to the occurrence and development of HCC. 10–100 μM NaHS promoted the migration and growth of HCC cells, while 600–1,000 μM NaHS had the opposite effect. 25–100 μM NaHS inhibited HCC apoptosis, while 400–1,000 μM NaHS had the opposite effect. These results indicated that the low concentration of H_2_S promoted HCC, while the high concentration of H_2_S inhibited HCC. Further research showed that 25–50 μM NaHS upregulated the protein levels of phosphorylated extracellular signal-regulated kinase (p-ERK), phosphorylated epidermal growth factor receptor (p-EGFR), matrix metalloproteinase-2 (MMP-2) and phosphorylated protein kinase B (p-AKT), and downregulated the ratio of Bax/Bcl-2 and the levels of phosphatase and tensin homolog (PTEN). While 800–1,000 μM NaHS had the opposite effect in HCC cells. Similar to that *in vitro*, the low concentration of H_2_S promoted the growth and angiogenesis of HCC xenografts in nude mice, while the high concentration of H_2_S had the opposite effect. These results indicated that the low concentration of H_2_S activated PTEN/AKT and EGFR/ERK/MMP-2 pathways, while the high concentration of H_2_S had the opposite effects (Wu et al., 2017). It has been reported that EGFR/ERK/MMP-2 and PTEN/AKT pathways contribute to the development of HCC (Qian et al., 2015; Yang et al., 2020; Wang et al., 2021b). Therefore, it can be deduced that exogenous H_2_S plays a double-edged sword role in HCC cells through regulating angiogenesis and apoptosis via EGFR/ERK/MMP-2 and PTEN/AKT pathways (Wu et al., 2017).

### 3.2 Endogenous H_2_S promotes hepatocellular carcinoma

CSE is a vitamin B6-dependent enzyme that catalyzes the production of endogenous H_2_S (Chiku et al., 2009). It is generally expressed in the liver, heart, kidney, ileum, pancreatic islet, placenta and vascular system, but not in the central nervous system (Kimura, 2010). The change in CSE expression is related to the change in the level of endogenous H_2_S, thus participating in the progress of various diseases such as cancer and diabetes (Jia et al., 2022; Omorou et al., 2022). The PI3K/Akt signal pathway is an important signal pathway regulating cell growth, proliferation, metabolism, survival, and movement (Akbarzadeh et al., 2021; Korkmaz et al., 2022). Many studies have demonstrated that the PI3K/AKT pathway regulates HCC (Li et al., 2021; Sun et al., 2021; Zhou et al., 2021). However, the relevant mechanisms are not completely understood. In addition, CSE is upregulated by the PI3K/AKT pathway (Wang et al., 2022a). Therefore, it can be speculated that CSE and PI3K/AKT pathways play a vital role in HCC. Peng Yin and colleagues found that the PI3K/AKT pathway positively regulated the expression of CSE in HCC cells. Akt deletion or PI3K inhibitor could reduce the expression of CSE, while Akt activation could upregulate CSE expression. The PI3K/AKT pathway regulated the expression of CSE at the transcriptional level. The double luciferase transporter analysis showed that the −592/+139 gene fragment was the core promoter of *CSE*. The specificity protein 1 (SP1) was an important transcription factor and could directly bind to the core promoter of CSE to regulate CSE expression. The mutation of the Sp1 binding core promoter of *CSE* reversed the PI3K/AKT pathway-induced expression of *CSE*, indicating that the PI3K/Akt pathway upregulated the expression of *CSE* through Sp1 binding to the core promoter of CSE. Moreover, the production of endogenous H_2_S was positively related to the expression of CSE, and CSE/H_2_S promoted HCC cell proliferation by inducing cell cycle progression. Collectively, the PI3K/Akt pathway upregulated the expression of CSE through Sp1 binding to the core promoter of *CSE*, thus promoting HCC, indicating that endogenous H_2_S promoted HCC(86). The Sp1 promoted the transcription of genes encoding cyclinD, p21Cip/WAK-1, and cyclin E, which were involved in cell cycle progression (Sherr and Roberts, 2004; Santiago et al., 2007). This is consistent with the conclusion of the above study that CSE/H_2_S promotes the growth of HCC cells by promoting cell cycle progression. Another study by Yan Pan et al. further clarified the mechanism of endogenous H_2_S promotion of HCC. The results revealed that CSE was upregulated in HCC cells. The inhibition of endogenous H_2_S/CSE pathway by propargylglycine (PPG)/CSE siRNA significantly reduced the proliferation of HCC cells, indicating that the H_2_S/CSE pathway induced the proliferation of HCC cells. In addition, the inhibition of the H_2_S/CSE pathway promoted ROS production, DNA damage and mitochondrial disruption, and upregulated the apoptosis of HCC cells. The increased apoptosis was related to the activation of p53 and p21, the decrease of the Bcl-2/Bax ratio, and the increase of caspase-3 and phosphorylated c-Jun N-terminal kinase (p-JNK) activity. Moreover, the suppression of the proliferation of HCC cells by the suppression of the H_2_S/CSE pathway was related to the inhibition of the epidermal growth factor receptor (EGFR) through suppressing extracellular signal-regulated kinase 1/2 (ERK1/2). Summarily, it could be deduced from the above that CSE/H_2_S promotes the proliferation of HCC cells by inhibiting mitochondrial ROS-mediated apoptosis through activating the EGFR/ERK1/2 pathway (Pan et al., 2014). ROS has been reported to induce apoptosis (Cui et al., 2021; Fontana et al., 2021). In the above study, H_2_S/CSE promotes HCC cell proliferation by inhibiting HCC cell apoptosis through suppressing ROS production (Pan et al., 2014).

H_2_S/CSE can not only promote HCC growth but also promote the metastasis of HCC. With the progress of technology, the accuracy of extracorporeal radiotherapy is getting higher and higher, which allows radiotherapy to be applied to patients with HCC (Chen et al., 2021). Although radiotherapy can significantly improve the survival rate of HCC patients, the metastasis and recurrence of HCC after radiotherapy are more common in clinical practice (Wang et al., 2022b). Therefore, it is particularly important to determine the factors that promote the metastasis of HCC cells after radiotherapy to improve the therapeutic effect of HCC. The study of Hang Zhang et al. showed that in xenograft tumors *in vivo*, both single-dose and fractionated irradiation promoted the metastasis of HCC cells 20–60 days after irradiation. Radiation upregulated the expressions of epithelial-mesenchymal transition (EMT) marker proteins including N-cadherin and Snail, and downregulated the E-cadherin expression *in vivo* and *in vitro*, suggesting that radiation-induced long-term EMT in HCC. The in-depth research revealed that in HCC cells treated with the single-dose irradiation, the expression levels of CSE and CBS, and the phosphorylation of p38 mitogen-activated protein kinases (MAPK) increased significantly, indicating that radiation upregulated endogenous H_2_S and p38 MAPK pathways. The inhibition of CSE or CBS, two endogenous H_2_S-producing enzymes, notably abolished the upregulated expressions of EMT marker proteins and p38 MAPK induced by radiation, indicating that H_2_S/CSE promoted EMT and p38 MAPK signaling pathways in HCC. Furthermore, the inhibition of p38 MAPK also abolished the radiation-induced expressions of EMT marker proteins, indicating that H_2_S/CSE promoted long-term metastasis of HCC cells after irradiation through promoting EMT by activating the p38 MAPK pathway, therefore inducing the invasion and metastasis of HCC cells and the xenograft tumors (Zhang et al., 2018). Contrary to the conclusion that H_2_S/CSE promoted EMT, exogenous H_2_S inhibited transforming growth factor beta (TGF β)-induced EMT of HCC cells (Fang et al., 2010; Guo et al., 2016). The above contradictory imagination may be related to the cell type and H_2_S concentration. Perhaps the low concentration of endogenous H_2_S promotes EMT of cells, while the high concentration of endogenous H_2_S has the opposite effect, which needs to be further studied.

## 4 Conclusion

**FIGURE 2:**
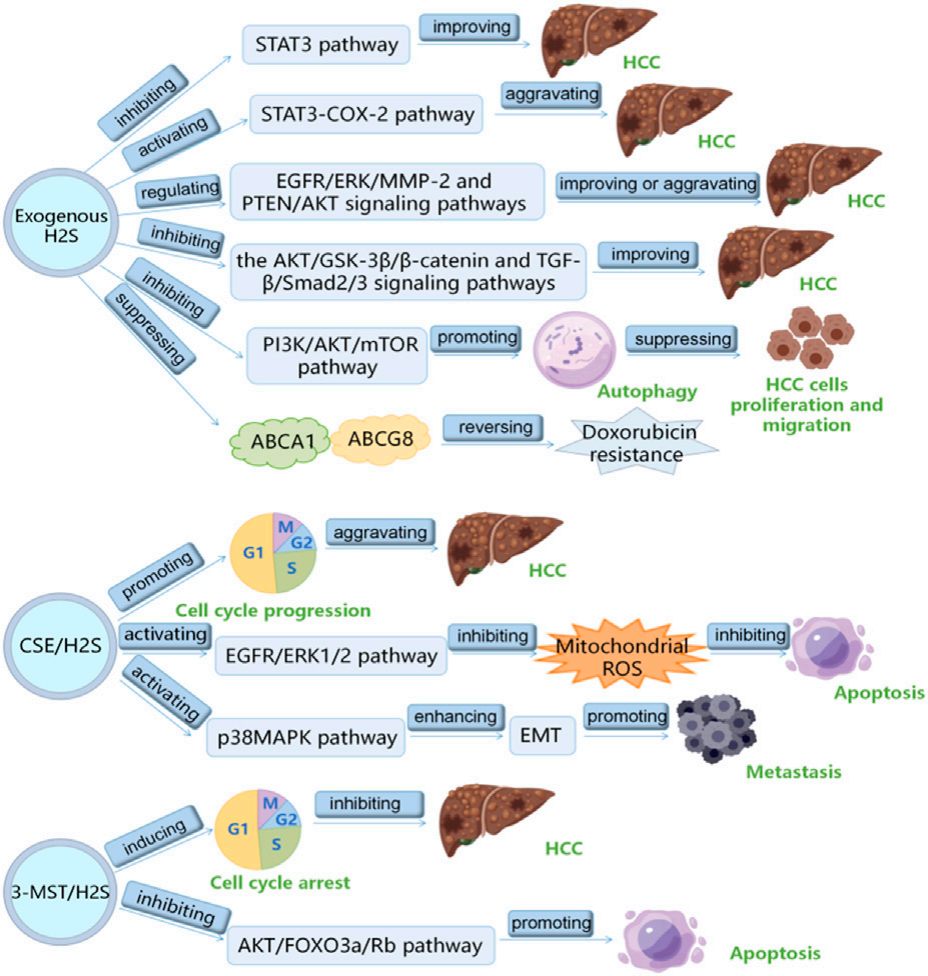
The summary of the role of hydrogen sulfide in hepatocellular carcinoma.

H_2_S plays a vital role in HCC, which has been the research hotspot recently. Here, we summarizes the role and mechanism of H_2_S in HCC as follows: 1) exogenous H_2_S improves HCC by inhibiting STAT3 pathway; 2) exogenous H_2_S aggravates HCC via inducing STAT3-COX-2 pathway; 3) exogenous H_2_S plays a double-edged sword role in HCC through PTEN/AKT and EGFR/ERK/MMP-2 pathways; 4) exogenous H_2_S suppresses HCC cells proliferation and migration by inducing autophagy via suppressing PI3K/AKT/mTOR pathway; 5) HA-ADT suppresses HCC cells through inhibiting TGF-β/Smad2/3 and AKT/GSK-3β/β-catenin pathways; 6) PI3K/Akt pathway increases CSE/H_2_S level through Sp1 binding to *CSE* core promoter to aggravate HCC through promoting the cell cycle progression; 7) CSE/H_2_S promotes the proliferation of HCC cells by inhibiting mitochondrial ROS-mediated apoptosis through activating EGFR/ERK1/2 pathway; 8) CSE/H_2_S promotes long-term metastasis of HCC cells after irradiation through enhancing EMT by activating the p38MAPK pathway; 9) 3-MST/H_2_S inhibits HCC through promoting the apoptosis and cell cycle arrest of HCC cells by inhibiting AKT/FOXO3a/Rb pathway; 10) exogenous H_2_S reverses doxorubicin resistance to HCC via inhibiting the expressions of ABCG8 and ABCA1 (Table 1) (Figure 2). It can be seen from the above that several signal pathways, including EGFR/ERK/MMP-2 pathway, STAT3-COX-2 pathway, PI3K/AKT/mTOR pathway, PTEN/AKT signaling pathway, p38 MAPK pathway and AKT/FOXO3a/Rb pathway, participate in the role and mechanism of H_2_S in HCC. In addition to the signal pathways mentioned in this review, it is worth further studying whether H_2_S can play a role in HCC through other signal pathways. Moreover, in this review, H_2_S regulates HCC by regulating the cell cycle, apoptosis, angiogenesis, doxorubicin resistance, metastasis, proliferation, and migration of HCC cells. Can H_2_S also play a role in HCC through other mechanisms? For example, H_2_S plays a role in HCC by regulating pyroptosis and ferroptosis. In addition, the cost of the H_2_S-related drugs is generally cheaper. If they can be used to treat HCC, the economic burden of HCC patients will be greatly reduced. However, the current research shows that H_2_S plays a dual role in cancers (such as HCC) and inflammation. Therefore, more research is needed in the future to clarify under what conditions H_2_S promotes HCC and under what conditions H_2_S has the opposite effect. Furthermore, it is particularly important to avoid the occurrence of side effects such as H_2_S promoting cancer and inflammation when H_2_S-related drugs are used to treat HCC patients in the future. At present, the exogenous H_2_S releaser has many limitations, such as being unable to maintain a high concentration of H_2_S for a long time. Therefore, it is urgent to find new long-acting H_2_S-releasing agents so that it is possible to apply H_2_S-related drugs to the clinical treatment of HCC.

**TABLE 1:** The summary of the role of hydrogen sulfide in hepatocellular carcinoma.

The role of hydrogen sulfide (H_2_S) in hepatocellular carcinoma (HCC)	Experimental model	References
Exogenous H_2_S improves HCC via inducing cell cycle arrest and apoptosis of HCC cells through inhibiting STAT3 pathway	HCC cell lines (HepG2 and Bel7402)/mice model of HCC	Lu et al. (2014)
Exogenous H_2_S suppresses HCC cells proliferation and migration through promoting autophagy by suppressing PI3K/AKT/mTOR pathway	HCC cell (HepG2 and HLE cells)	Wang et al. (2017)
Exogenous H_2_S reverses doxorubicin resistance to HCC by suppressing the expressions of ABCA1 and ABCG8	HCC cell lines (HepG2 cells)	Stokes et al. (2018)
HA-ADT inhibited HCC cells via promoting apoptosis via suppressing the AKT/GSK-3β/β-catenin, and inhibiting autophagy through suppressing TGF-β/Smad2/3 signaling pathways	HCC cell lines SMMC-7721 and Huh-7 and mice model of HCC	Duan et al. (2023)
3-MST/H_2_S inhibits HCC through promoting the cell cycle arrest and apoptosis of HCC cells by inhibiting AKT/FOXO3a/Rb pathway	Human HCC cell lines (HepG2, MHCC-LM3, Huh7 and Hep3B)and samples from HCC patients	Li et al. (2022b)
Exogenous H_2_S aggravates HCC by activating the STAT3-COX-2 pathway	HCC cell lines (PLC/PRF/5 cells)	Zhen et al. (2018)
Exogenous H_2_S plays a double-edged sword role in HCC via EGFR/ERK/MMP-2 and PTEN/AKT signaling pathways	HCC cell lines (SMMC-7721 and Huh-7)	Wu et al. (2017)
PI3K/Akt pathway increases the level of CSE/H_2_S through Sp1 binding to the core promoter of *CSE* to aggravate HCC through promoting the cell cycle progression	HCC cell lines (QGY-7703 and SMMC-7721)	Yin et al. (2012)
CSE/H_2_S promotes the proliferation of HCC cells by inhibiting mitochondrial ROS-mediated apoptosis through activating EGFR/ERK1/2 pathway	HCC cell lines (HepG2, PLC/PRF/5, Hep3B cells)	Pan et al. (2014)
CSE/H_2_S promotes long-term metastasis of HCC cells after irradiation through enhancing EMT by activating the p38MAPK pathway	HCC cell lines (HepG2 cells)	Zhang et al. (2018)

We believe that the H_2_S-related drugs will become a new strategy for HCC treatment.
